# Comment on: ‘Antibiotic footprint’ as a communication tool to aid reduction of antibiotic consumption

**DOI:** 10.1093/jac/dkz251

**Published:** 2019-06-15

**Authors:** Dominic Moran

**Affiliations:** Global Academy of Agriculture and Food Security, The University of Edinburgh, The Royal (Dick) School of Veterinary Studies and The Roslin Institute, Alexander Robertson Building, Easter Bush Campus, Midlothian, UK

Sir,

In an interesting contribution, Limmathurotsakul *et al*.^1^ suggest that an antibiotic footprint calculation analogous to a more familiar carbon footprint may be a compelling way to represent the different forms of antibiotic use and to benchmark performance in reducing use across clinical and agricultural settings. In doing so, they are the latest of several papers highlighting the parallels between metrics used to manage climate change mitigation (i.e. reduction in greenhouse gas emissions) and antibiotic use as a precursor to antimicrobial resistance (AMR).^2–4^

However, it is important to be clear on what a footprint concept represents and to consider that its application to antibiotics may potentially be misleading. Originally, the ecological footprint signifies the resource requirement to offset ecological damage,^5^ typically the area of land required to offset the greenhouse gas emissions of a spatial jurisdiction. Thus, the UK footprint could be equated to an area required to plant trees to sequester the annual emission of CO_2_ equivalent. Going beyond the greenhouse gas metric alone, another eye-catching interpretation suggests that humanity uses the equivalent of 1.7 Earths to provide the resources we use and absorb our waste. In other words, the globe has an ecological deficit and the calculation can even pinpoint the day of the year when this starts.

Footprints provide a compelling message to governments and consumers about the sustainability or otherwise of current consumption trends (e.g. there is no planet B) and are a precursor to literature on planetary boundaries.^6^ This is a bit more sophisticated than counting the impact, e.g. total life-cycle emissions of a production process as apparently implied by Limmathurotsakul *et al*.,^1^ who seemingly equate the footprint to an alternative representation of antimicrobial use inventory data.

There are several potential policy-relevant metrics to represent antibiotic use and it is important to be clear on their interpretation in the AMR context. We should also avoid overstating the power of footprinting rhetoric, when evaluation evidence is conspicuously absent. More importantly, apart from drug innovation, there is no obvious offsetting analogy applicable to AMR. It is important that this message is clear in any metric.

## Funding

The author acknowledges support from UK Newton Fund awards co-funded by the Economic and Social Research Council grant numbers ES/S000186/1 and ES/S000208/1, linked to the India Department of Biotechnology grant numbers BT/IN/Indo-UK/AMR/06/BRS/2018-19 and BT/IN/Indo-UK/AMR/03/RKE/2018-19.

## Transparency declarations

None to declare.
